# Detecting *Escherichia coli* Contamination on Plant Leaf Surfaces Using UV-C Fluorescence Imaging and Deep Learning

**DOI:** 10.3390/plants14213352

**Published:** 2025-10-31

**Authors:** Snehit Vaddi, Thomas F. Burks, Zafar Iqbal, Pappu Kumar Yadav, Quentin Frederick, Satya Aakash Chowdary Obellaneni, Jianwei Qin, Moon Kim, Mark A. Ritenour, Jiuxu Zhang, Fartash Vasefi

**Affiliations:** 1 Department of Computer and Information Science and Engineering, University of Florida, Gainesville, FL 32611, USA; 2 Department of Agricultural and Biological Engineering, University of Florida, Gainesville, FL 32611, USA; 3 Department of Agricultural and Biosystems Engineering, South Dakota State University, Brookings, SD 57007, USA; 4 USDA/ARS Environmental Microbial and Food Safety Laboratory, Beltsville Agricultural Research Center, Beltsville, MD 20705, USA; 5 Department of Horticultural Sciences, University of Florida, 2199 South Rock Road, Fort Pierce, FL 34945, USA; 6 SafetySpect Inc., 4200 James Ray Dr., Grand Forks, ND 58202, USA

**Keywords:** *E. coli*, food safety, fluorescence imaging, CSI-D+, deep learning, YOLO11, Eigen-CAM

## Abstract

The transmission of *Escherichia coli* through contaminated fruits and vegetables poses serious public health risks and has led to several national outbreaks in the USA. To enhance food safety, rapid and reliable detection of *E. coli* on produce is essential. This study evaluated the performance of the CSI-D+ system combined with deep learning for detecting varying concentrations of *E. coli* on citrus and spinach leaves. Eight levels of *E. coli* contamination, ranging from 0 to 10^8^ colony-forming units (CFU)/mL, were inoculated onto the leaf surfaces. For each concentration level, 10 droplets were applied to 8 citrus and 12 spinach leaf samples (2 cm in diameter), and fluorescence images were captured. The images were then subdivided into quadrants, and several post-processing operations were applied to generate the final dataset, ensuring that each sample contained at least 2–3 droplets. Using this dataset, multiple deep learning (DL) models, including EfficientNetB7, ConvNeXtBase, and five YOLO11 variants (n, s, m, l, x), were trained to classify *E. coli* concentration levels. Additionally, Eigen-CAM heatmaps were used to visualize the spatial responses of the models to bacterial presence. All YOLO11 models outperformed EfficientNetB7 and ConvNeXtBase. In particular, YOLO11s-cls was identified as the best-performing model, achieving average validation accuracies of 88.43% (citrus) and 92.03% (spinach), and average test accuracies of 85.93% (citrus) and 92.00% (spinach) at a 0.5 confidence threshold. This model demonstrated an inference speed of 0.011 s per image with a size of 11 MB. These findings indicate that fluorescence-based imaging combined with deep learning for rapid *E. coli* detection could support timely interventions to prevent contaminated produce from reaching consumers.

## 1. Introduction

From 2010 to 2024, a total of 192 national outbreaks were reported in the US, caused by several pathogens that led to severe health issues, including hemorrhagic colitis, gastroenteritis, and even death. Among these, *E. coli* strains contributed to the most outbreaks (22.68%), followed by Listeria monocytogenes (17%). Specifically, *E. coli O157:H7* alone accounted for approximately 12.89% of the outbreaks, making *E. coli* strains the dominant group overall [1].

In terms of food sources, 28.6% of outbreaks were linked to vegetables and leafy greens, 26.6% to meat products, 9.9% to dairy products, and 34.9% to other foods. *E. coli* strains were responsible for 43.2% of outbreaks associated with vegetables and leafy greens, 18.2% with meat products, and 6.8% with dairy products [1]. Fruits, vegetables and leafy greens are especially susceptible to contamination during harvesting, postharvest handling, and processing due to direct contact with contaminated water, soil, or equipment. This is exacerbated by the minimal processing steps typically applied to these products, thereby increasing the risk of pathogen survival. As a result, there is an urgent need for rapid and non-invasive detection techniques that can identify *E. coli* on leaf surfaces, prevent contaminated products from reaching consumers, and strengthen food safety protocols.

The combination of type C ultraviolet light (UV-C) and fluorescence imaging has emerged as a promising tool for the rapid and non-invasive detection of microbial biofilms, such as those formed by bacteria and fungi, on organic surfaces [2,3,4]. When exposed to UV-C wavelengths (typically 254–280 nm), certain biomolecules in bacterial cells, such as aromatic amino acids (e.g., tryptophan and tyrosine) and coenzymes, emit characteristic fluorescence [5,6]. This range includes low-pressure mercury lamps at 254 nm and germicidal LEDs peaking between 265 and 280 nm, representing the most common UV-C sources for microbial detection. This fluorescence-based approach offers surface-level sensitivity, making it well-suited for detecting *E. coli* on produce, as the bacteria may be introduced through irrigation water, soil, or handling during postharvest operations [7]. Unlike conventional methods such as culturing or PCR, UV-C imaging provides immediate feedback without the need for sample preparation or destruction, allowing for real-time monitoring in food processing environments [8,9,10]. However, culturing detects viable microorganisms and PCR detects nucleic acids, while UV-C imaging detects surface fluorescence, reflecting different targets and preventing direct comparison of detection limits. Furthermore, the integration of fluorescence imaging with artificial intelligence and deep learning techniques enhances detection accuracy by classifying fluorescence patterns associated with varying levels of contamination [11]. To automatically detect *E. coli* in microscopic images, three deep learning object detection networks (SSD-MobileNetV2, EfficientDet, and YOLOv4) were evaluated. Among them, YOLOv4 performed best, achieving a mean average precision (mAP) of 98% [12]. A review article demonstrated that in several studies, convolutional neural networks (CNNs) and transfer learning were used on laboratory-prepared datasets to distinguish bacterial species, including foodborne pathogens and antibiotic-resistant strains, with accuracies ranging from 81% to 100% [13]. In another study, a VGG16-based network was employed with transfer learning, fine-tuning, and data augmentation to classify superficial fungal infections, reporting an accuracy improvement from 84.38% initially to 97.19% after augmentation [14]. A mAP of 91.64% was achieved in a study during the detection of fungus from fluorescence images using the YOLOv8 framework [4]. Recently, another workflow using fluorescence images and based on YOLOv8 was developed to detect surface contamination under variable ambient lighting conditions, achieving a mAP of 69.2% under optimal conditions and 62.2% under noisy conditions [15]. Likewise, several deep learning algorithms, including CNNs, Generative Adversarial Networks (GANs), and Recurrent Neural Networks (RNNs), have been successfully utilized to detect bacterial and fungal concentration levels on organic surfaces using fluorescence imaging [16].

It was found that the DL algorithms performed significantly better when the fluorescence images were denoised prior to training the algorithm [16]. Raw fluorescence images are often affected by various sources of noise, such as sensor artifacts, tissue autofluorescence, and uneven illumination, which can obscure biologically relevant signals. While basic background removal techniques target average noise levels, they are often insufficient to handle structured noise caused by spatially varying illumination or autofluorescence. These complex artifacts introduce a range of spatial frequencies that simple background subtraction cannot eliminate, potentially leading to misinterpretation by the model [17]. Advanced denoising methods are therefore necessary to enhance the signal-to-noise ratio (SNR), preserve meaningful features, and provide clean, high-quality inputs that improve model accuracy and robustness [18].

Several advanced denoising approaches, such as wavelet filtering, non-local means, Discrete Wavelet Transform (DWT), and DL-based denoisers, have proven effective in preserving key spatial features while suppressing noise. Among these, Discrete Wavelet Transform (DWT) has emerged as one of the effective techniques due to its ability to preserve both spatial and frequency information simultaneously [19]. DWT is a multiresolution analysis method that decomposes images into different frequency bands, separating coarse approximations from fine details using a combination of low-pass and high-pass filters [20]. This decomposition enables selective noise suppression, as most noise is concentrated in the high-frequency sub-bands. Thresholding techniques such as soft, hard, and adaptive thresholding can be applied to these coefficients to suppress noise while preserving image features [21,22,23]. Hupfel et al. [24] demonstrated that DWT significantly improved the visibility of intracellular structures in noisy confocal fluorescence images, outperforming traditional spatial-domain filters. Similarly, a study by Wang et al. [25] showed that DWT-denoised fluorescence images enhanced the accuracy of DL methods for the restoration of microscopic images. In another study, Xiangyu et al. [26] used DWT-based preprocessing before feeding the images into a CNN, resulting in improved image classification. In agricultural applications, fluorescence imaging combined with wavelet denoising has been used to identify contamination on fruit surfaces [4]. In these cases, DWT enhanced the contrast of biological features and removed structured background noise from uneven UV excitation, thereby improving the signal quality before analysis [27,28].

While denoising improves signal quality and model performance, understanding the predictions of DL models is important. However, characterizing these models by numerous layers and parameters is a challenging task due to their complexity. Models like YOLO operate as black-box solutions, and directly interpreting each computation is not feasible. Therefore, the utilization of Explainable AI (XAI) tools becomes essential to extract and translate information from various layers into interpretable insights [29]. One such advanced technique, the Eigen-Class Activation Map (Eigen-CAM) [30], provides a class-agnostic visualization of influential regions in an image by analyzing the principal components of feature maps, thus aiding in understanding model decisions. This technique has been applied in the training and interpretation of various DL models across diverse research domains [31]. For instance, it has been utilized to localize pathological regions in medical imaging [32,33], highlight key visual features for object recognition in autonomous driving systems [34,35], and assist in classification and anomaly detection tasks in agricultural applications [36,37].

Building on prior research and addressing the lack of rapid, on-leaf quantification of *E. coli* concentrations, this study aimed to develop a robust and explainable DL-based workflow using a fluorescence-based, handheld 275 nm UV-C imaging system. The specific objectives of this research were as follows:

- Generate a fluorescence imaging dataset of E. coli on two distinct leaf surfaces at four concentration levels, ranging from 0 to 108 CFU/mL, using the SafetySpect CSI-D+ system.
- Construct a processing pipeline that includes image preparation, data augmentation, denoising, and training of multiple DL classifiers for accurate classification of E. coli concentration levels.
- Validate the proposed workflow using independent datasets to assess its accuracy and robustness and utilize Eigen-CAM visualizations to interpret model predictions and highlight key regions influencing classification outcomes.

## 2. Results and Discussion

### 2.1. Image Denoising

**Figure 1:**
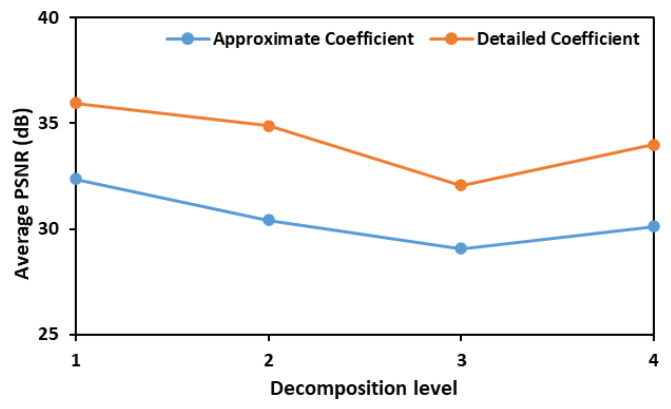
Test results showing average PSNR values that were obtained when denoising fluorescence images consisting of *E. coli* concentration at all the eight different levels.

In the initial phase of this study, both detailed and approximate coefficients were assessed to calculate PSNR values and identify the most effective wavelet transform for denoising the fluorescence images of leaves inoculated with different concentrations of *E. coli*. Figure 1 illustrates the average Peak Signal-to-Noise Ratio (PSNR) values obtained using six different wavelet functions—bior1.3, coif1, db3, haar, rbio1.3, and sym2—across various decomposition levels. The PSNR values are plotted against the decomposition levels for both approximate (low-frequency) and detailed (high-frequency) coefficients. The results indicate that a decomposition level of 1 yields the highest PSNR values for both coefficient types, with the detailed coefficients consistently exhibiting higher average PSNR values than the approximate coefficients. This suggests that retaining finer details at the initial decomposition level is crucial for optimal denoising.

**Figure 2:**
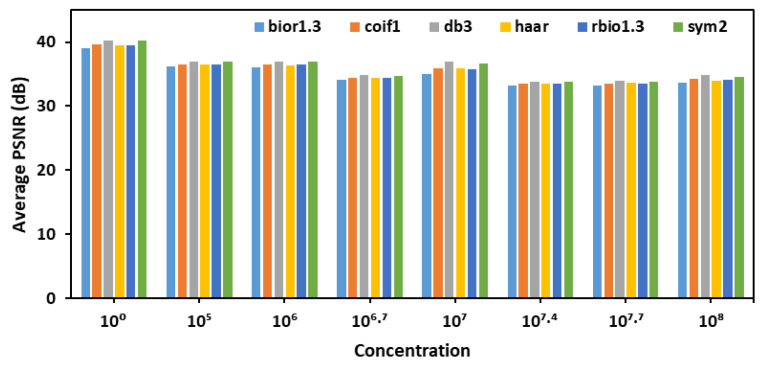
Average PSNR values for detailed coefficients across six wavelet transforms evaluated for denoising fluorescence images of leaves.

After selecting detailed coefficients for calculating PSNR and choosing decomposition level 1 for denoising, six different wavelet transforms- bior1.3, coif1, db3, haar, rbio1.3, and sym2- were evaluated across various *E. coli* concentration levels as shown in Figure 2. The goal was to identify the most effective wavelet for denoising fluorescence images of the leaves. The analysis, based on average PSNR (Peak Signal-to-Noise Ratio) values, revealed that the db3 wavelet consistently achieved the highest PSNR values across different concentrations, indicating superior performance in preserving image quality during decomposition and reconstruction.

Despite its effectiveness, the wavelet transform approach had some limitations. The choice of wavelet function and decomposition level significantly affected denoising performance and may not generalize well to all image types or noise characteristics. Additionally, wavelet-based denoising sometimes oversmoothed fine features or introduced artifacts, especially at higher decomposition levels. Future work could explore hybrid or adaptive denoising methods to address these limitations and further improve image quality.

### 2.2. Performance Evaluation of YOLO11 Models

After finalizing the decomposition level, selecting the optimal wavelet method, and applying image augmentation, five YOLO11 variants (n, s, m, l, and x) were evaluated for classifying citrus and spinach leaves across eight levels of *E. coli* concentration. Three dataset configurations (8 classes, 8 classes and 4 bins, and 4 classes) were used to assess performance based on validation accuracy, test accuracy, and inference time.

**Figure 3:**
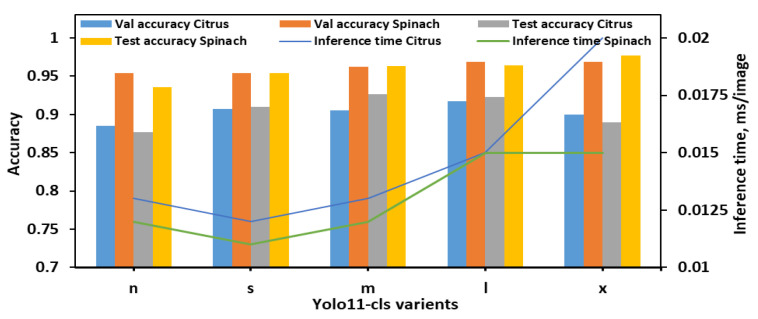
Performance metrics of all YOLO11 variants for classifying *E. coli* concentration levels in citrus and spinach leaves using 8classes dataset.

During the evaluation using 8classes dataset (Figure 3), YOLO11n-cls exhibited the lowest performance, with test accuracies of 0.877 and 0.936 for citrus and spinach leaves, respectively, despite achieving the fastest inference times (0.013 s and 0.012 s). YOLO11s-cls showed improved accuracy for both datasets (0.910 for citrus and 0.954 for spinach) while maintaining a low inference time, comparable to YOLO11n-cls. The medium-sized model, YOLO11m-cls, achieved a favorable balance between classification performance and computational efficiency, attaining test accuracies of 0.926 for citrus and 0.963 for spinach, with inference times of 0.013 s and 0.012 s, respectively. YOLO11l-cls provided the highest validation accuracies for both citrus (0.917) and spinach (0.969), and comparable test accuracies (0.923 for citrus and 0.964 for spinach), though it required slightly longer inference times (0.015 s for both datasets). Notably, YOLO11x-cls achieved the highest spinach test accuracy (0.977), but its performance on citrus data decreased (0.890), and it incurred the longest inference time of all models (0.020 s for citrus). Overall, YOLO11m-cls was identified as the most effective model for 8classes datasets, offering consistent and high classification performance across both leaf samples with minimal computational overhead. Although YOLO11x-cls outperformed other models on the spinach dataset in terms of test accuracy, its increased inference time and reduced citrus accuracy suggested limited generalizability.

**Figure 4:**
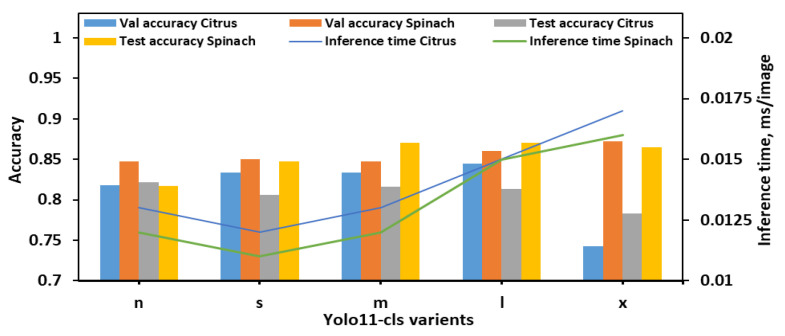
Performance metrics of all YOLO11-cls variants for classifying *E. coli* concentration levels in citrus and spinach leaves using 8classes_4bins dataset.

With the 8classes_4bins dataset, overall performance trends differed slightly from those observed in the 8classes dataset as illustrated in Figure 4. The YOLO11n-cls model achieved moderate accuracy with relatively fast inference times, recording test accuracies of 0.822 for citrus and 0.817 for spinach, and inference times of 0.013 s and 0.012 s, respectively. The YOLO11s-cls model performed slightly better on spinach (test accuracy of 0.847) but showed a drop in citrus test performance (0.806), despite being the fastest model (0.012 s and 0.011 s for citrus and spinach, respectively). YOLO11m-cls delivered a balanced performance, achieving validation accuracies of 0.834 for both citrus and spinach, and improved test accuracy for spinach (0.870), although its performance on citrus remained moderate (0.816). The YOLO11l-cls model attained the highest validation accuracy for citrus (0.845) and a strong value for spinach (0.860), with test accuracies of 0.813 for citrus and 0.870 for spinach. While its inference time (0.015 s for both datasets) was higher than smaller models, it remained computationally reasonable. YOLO11x-cls achieved the best validation accuracy for spinach (0.872) and strong test accuracy (0.865), but its performance on citrus dropped considerably (validation: 0.743; test: 0.783), with inference times of 0.017 s and 0.016 s for citrus and spinach, respectively. Considering all metrics, YOLO11m-cls and YOLO11l-cls demonstrated the most stable and accurate performance across both leaf types in the 8classes_4bins dataset. YOLO11m-cls was especially notable for achieving high spinach test accuracy with a low computational cost, while YOLO11l-cls offered slightly improved validation metrics at the expense of a marginally longer inference time. Although YOLO11x-cls performed well on spinach, its significantly lower accuracy on citrus and higher computational demands reduced its overall reliability. Therefore, for the 8cls_4Bucket dataset, YOLO11m-cls emerged as the most balanced and efficient model, while YOLO11l-cls offered the best validation accuracy for citrus with comparable performance on spinach.

**Figure 5:**
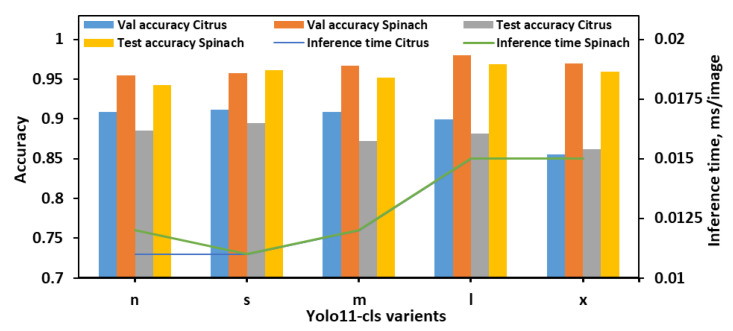
Performance metrics of all YOLO11-cls variants for classifying *E. coli* concentration levels in citrus and spinach leaves using 4classes dataset.

The classification performance of the YOLO11-cls models was evaluated on the 4classes dataset, which consisted of the citrus and spinach leaves categorized into four *E. coli* concentration levels (Figure 5). Across all models, spinach samples consistently achieved higher validation and test accuracies than citrus, indicating potentially more distinguishable class features in spinach leaves. The YOLO11n-cls model achieved validation accuracies of 0.909 and 0.955 for citrus and spinach, respectively, and corresponding test accuracies of 0.885 and 0.942. It also recorded the fastest inference times (0.011 s for citrus and 0.012 s for spinach), making it a lightweight and computationally efficient option. YOLO11s-cls marginally outperformed YOLO11n-cls in both validation and test accuracies, achieving the highest citrus test accuracy (0.895) and the second-highest spinach test accuracy (0.961), while maintaining the lowest inference time (0.011 s for both datasets). YOLO11m-cls demonstrated strong validation accuracy on spinach (0.967) and good test accuracy (0.952), but its performance on citrus was slightly lower, with a test accuracy of 0.872. YOLO11l-cls achieved the highest validation accuracy for spinach (0.980) and strong test accuracy (0.969), indicating robust performance on spinach leaves. However, its citrus test accuracy (0.882) was slightly lower than YOLO11s-cls. YOLO11x-cls followed a similar trend, achieving high spinach validation (0.970) and test (0.959) accuracies, but its citrus performance remained the lowest among all models (validation: 0.855; test: 0.862), and it also incurred longer inference times (0.015 s for both datasets). Overall, YOLO11s-cls offered the best balance of performance and efficiency, attaining the highest citrus test accuracy and competitive spinach performance with minimal computational overhead. While YOLO11l-cls and YOLO11x-cls excelled on the spinach dataset, their increased inference times and slightly lower citrus accuracy reduced their suitability as general-purpose models for both leaf types. Therefore, YOLO11s-cls was identified as the optimal model for the 4classes dataset, providing strong generalization across both citrus and spinach datasets with the fastest inference speed.

Among the three datasets, 4classes enabled all models to achieve their highest average performance, likely due to its simplified class structure and clearer inter-class boundaries. Although larger models such as YOLO11l-cls and YOLO11x-cls performed exceptionally well on spinach in the 4classes dataset, their longer inference times and less stable performance on citrus reduced their overall practicality. In contrast, YOLO11s-cls and YOLO11m-cls consistently provided the best balance between accuracy and inference time across all datasets, achieving high classification performance for both citrus and spinach while maintaining computational efficiency. While YOLO11m-cls outperformed YOLO11s-cls by 0.22% in average validation accuracy and 0.48% in test accuracy, YOLO11s-cls offered an 8.83% shorter inference time and was 47.37% smaller in model size. Considering the intended deployment on edge devices, YOLO11s-cls was selected as the optimal model in this study, accepting the small trade-off in accuracy for its significant advantages in speed and lightweight architecture.

### 2.3. Comparative Performance: YOLO11 vs. Others

The best-performing variant of YOLO11 was further compared with other models trained to classify leaves with different *E. coli* concentration levels. In this study, the YOLO11s-cls model consistently outperformed EfficientNetB7 and ConvNeXtBase across all three datasets: 8classes, 8classes_4bins, and 4classes. For instance, YOLO11s-cls achieved the highest validation and test accuracies of 0.912 and 0.959, respectively, in the 4classes dataset. In contrast, the best test accuracies achieved by EfficientNetB7 and ConvNeXtBase on the same dataset were 0.763 and 0.840, respectively. In addition to its strong performance, YOLO11s-cls maintained a significantly smaller model size (11.0 MB) compared to EfficientNetB7 (254.7 MB) and ConvNeXtBase (338.1 MB), making it particularly suitable for deployment in resource-constrained environments. These results demonstrate that the YOLO-based architecture not only delivered superior classification accuracy but also offered considerable practical advantages in terms of model size and computational efficiency. A summary of these results is presented in Table 1.

### 2.4. Error Mode Analysis

**Figure 6:**
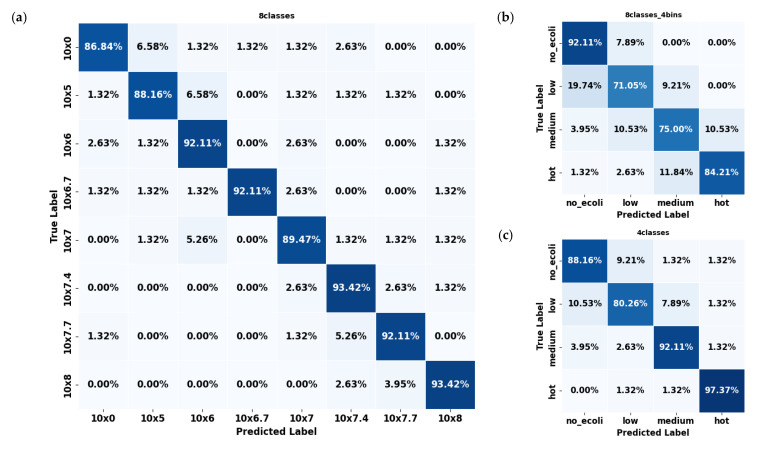
Confusion matrices of the YOLO11s-cls model used to classify *E. coli* concentration levels in citrus leaves under three dataset configurations: (**a**) 8classes, (**b**) 8classes_4bins, and (**c**) 4classes.

In evaluating the error mode of the best-performing model (YOLO11s-cls) for classifying *E. coli* concentrations in citrus and spinach leaves, the three dataset formats revealed distinct classification behaviors and error patterns. For citrus samples, the 8classes dataset delivered the highest overall accuracy (Figure 6a), with most classes classified correctly above 90%; however, slight confusion existed between adjacent concentration levels such as 10^6^, 10^6.7^, and 10^7^ CFU/mL. This pattern of minor spillover was expected due to the gradual progression of concentration levels, resembling regression tasks. The most significant misclassification observed reached approximately 6.58%, but these errors were largely confined to neighboring classes rather than random or distant misclassifications. The 8classes_4bins (Figure 6b) dataset reduced classification granularity but introduced broader errors, including a 19.74% misclassification rate of ‘low’ samples as ‘no_ecoli’ and a 10.53% confusion between ‘medium’ and ‘hot’. The 4classes (Figure 6c) dataset mitigated some of these issues, showing an 89.50% average accuracy and improved balance among categories, although over 10% of ‘low’ samples were still confused with ‘no_ecoli’.

**Figure 7:**
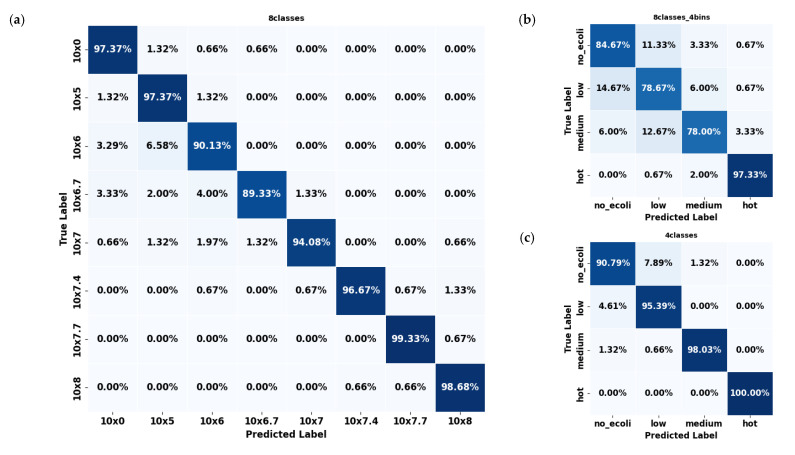
Confusion matrices of the YOLO11s-cls model used to classify *E. coli* concentration levels in spinach leaves under three dataset configurations: (**a**) 8classes, (**b**) 8classes_4bins, and (**c**) 4classes.

In contrast, spinach datasets consistently outperformed citrus across all configurations. The 8classes (Figure 7a) dataset showed high precision (≈94.50%), with errors mainly concentrated in mid-level concentrations such as 10^6^ and 10^5^. Multiple classes, including 0, 10^6^, and 10^6.7^, exhibited classification accuracy exceeding 96%. As with citrus, the few errors that did occur were predominantly between neighboring classes, reflecting a similar adjacent-class confusion pattern. However, the overall error rates were notably lower than in citrus, underscoring the model’s stronger discriminative ability for spinach samples across these eight concentration levels. The 8classes_4bins (Figure 7b) spinach dataset experienced some confusion, particularly between the ‘low’ and ‘medium’ categories, though still less severe than in citrus. The 4classes (Figure 7c) spinach dataset demonstrated outstanding performance, with every class exceeding 90% accuracy, most approaching or surpassing 98%, and the ‘hot’ class achieved a perfect classification (100%). Errors in this configuration were negligible, with confusion between ‘low’ and ‘medium’ limited to around 1.3%.

Overall, spinach datasets consistently exhibited fewer classification errors than citrus datasets across all configurations. This suggests that the YOLO11s-cls model was more effective at learning and distinguishing *E. coli* concentration levels in spinach leaves. Considering all configurations across both leaf types, the 4classes dataset provided the most consistent and least error-prone performance. The reduced complexity and broader classification bins of this division appeared to support better generalization, especially for spinach samples, where it surpassed all other datasets in accuracy and produced the cleanest confusion matrices.

Looking at the details of the best-performing YOLO11s-cls model (Table 2), it was observed that it achieved the highest performance on the 4classes dataset, with precision, recall, and F1-scores ranging from 0.894 to 0.961 for both citrus and spinach leaf samples. The 8classes dataset exhibited slightly lower metrics, while the 8classes dataset with 4bins grouping showed further reductions in performance (0.805–0.848). These results indicate that the model performed better on datasets with fewer or more consolidated classes, suggesting that simpler or more balanced class groupings enhanced its ability to accurately detect varying *E. coli* concentrations across different leaf types. It is important to note that performance evaluation was conducted with five different random seeds, yielding similar performance with a negligible standard deviation (<0.005). Therefore, only the mean values are reported.

### 2.5. Visualizing the Classified Images

**Figure 8:**
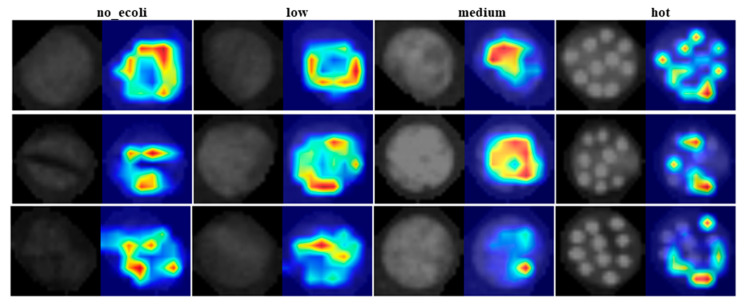
Eigen-CAM generated heat maps (**right**) for test denoised fluorescence images (**left**) consisting of *E. coli* at four different concentration levels on leaf surfaces.

Although the model was initially trained and tested on image quadrants containing 2–3 droplets of *E. coli* at each concentration level per sample, it was subsequently evaluated on full images containing all 10 droplets to assess its real-world applicability. The classification behavior of YOLO11 was visually interpreted using heat maps generated by Eigen-CAM, applied to test fluorescence images of *E. coli* inoculated leaf samples at four different concentration levels (Figure 8). These heat maps highlight the regions of the image that most strongly influenced the model’s decision making. It is important to clarify that the intensity of the heat map does not represent the actual *E. coli* concentration present in the sample. Instead, it indicates the spatial contribution, meaning the areas of the image that were most critical in determining the class label. In other words, the ‘hotter’ areas (regions with stronger activation) had greater influence on the model’s classifications, but do not necessarily correlate with greater *E. coli* presence. Crucially, these high activation regions are spatially aligned with the leaf structure. This alignment is evident when comparing the left sub-image (original fluorescence image) and the right sub-image (heat map overlay). The heat map activations consistently fall within the leaf boundaries, reinforcing that the model focused on biologically relevant regions rather than background or noise when making its predictions. This correspondence supports the reliability of the model’s attention mechanism and the interpretability of its decisions.

## 3. Materials and Methods

### 3.1. Workflow Pipeline

**Figure 9:**
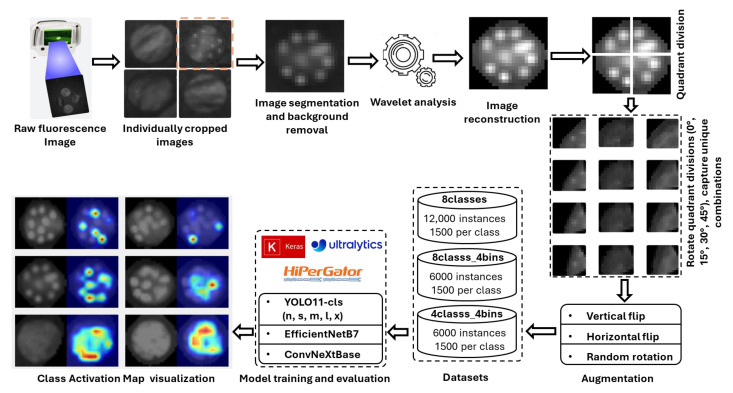
Workflow pipeline for *E. coli* concentration classification on plant leaf surfaces, illustrating image preprocessing, classification, and explainability using class activation map visualization.

The overall workflow pipeline employed to achieve the objectives of this study is illustrated in Figure 9. Initially, several circular areas (2 cm in diameter) were identified on spinach and citrus leaves. These areas were then inoculated with eight distinct *E. coli* concentration levels (0, 10^5^, 10^6^, 10^6.7^, 10^7^, 10^7.4^, 10^7.7^, and 10^8^ CFU/mL). Fluorescence images of these leaves were then captured using the CSI-D+ system with 275 nm UV-C illumination. The working distance from the imaging lens to the leaf was 153 mm for spinach (gain = 189, exposure = 70) and 150 mm for citrus leaves (gain = 164, exposure = 41). Although the irradiance at the leaf surface was not explicitly measured, the gain and exposure settings were adjusted for each leaf type to maximize the *E. coli* fluorescence without inducing autofluorescence from the leaf background. It was identified that the droplets deposited in the leaves were difficult to isolate in the captured images. Hence, images underwent denoising via wavelet transform [38], with the optimal wavelet determined through experiments measuring the average Peak Signal-to-Noise Ratio (PSNR) across all concentrations. PSNR was employed as a relative indicator of signal enhancement between processed and unprocessed images. All images were denoised using the wavelet transform that yielded the highest average PSNR for all concentrations. The denoised images were then augmented to expand the dataset, which was partitioned into training, validation, and testing subsets for the classification models. In the final stage, Eigen-CAM was applied to a selection of test images to generate heatmaps, providing visual explanations for the model’s predictions. The details of the workflow pipeline are further explained part by part in the following sections of this article.

### 3.2. E. coli Cell Preparation and Inoculation

**Figure 10:**
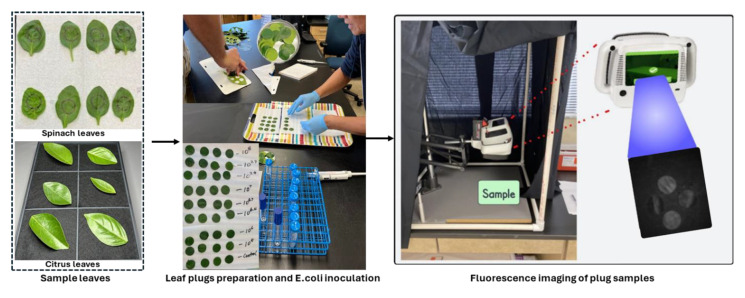
Sample preparation by inoculating *E. coli* bacterial colonies at eight different concentration levels on spinach and citrus leaf samples, followed by fluorescence image collection using the CSI-D+ system.

A non-pathogenic *E. coli* strain (ATCC 35218) was used as the target bacterium in this investigation, as it has previously been validated as an effective surrogate for *Salmonella spp.* in grapefruit studies [39]. The initial step involved streaking the *E. coli* strain onto tryptic soy agar medium followed by incubation at 35 °C for 24 h. A single colony from the resultant culture was then aseptically transferred into 10 mL of tryptic soy broth (TSB) contained within a test tube, and subsequent incubation was conducted at 35 °C for an additional 24 h. Following this incubation period, 10 µL of the *E. coli* suspension was sub-cultured into another 10 mL of TSB medium and incubated similarly for 24 h at 35 °C. The resulting *E. coli* suspension was subjected to centrifugation at 6000 rpm for 5 min, following which the supernatant was discarded, and the pellet containing the *E. coli* cells was resuspended in sterilized distilled water. This resuspension process was repeated once. Under these standardized conditions, the concentration of *E. coli* cells reached approximately 10^9^ CFU/mL. The actual concentration of *E. coli* cells in the suspensions was confirmed through serial dilution, plating, incubation, and subsequent enumeration of CFU. Various required concentrations of *E. coli*, such as 10^5^, 10^6^, 10^6.7^, 10^7^, 10^7.4^, 10^7.7^, and 10^8^ CFU/mL, were achieved through further dilution of the initial stock suspension (~10^9^ CFU/mL). Aliquots of *E. coli* cell suspensions at varying concentrations were deposited onto spinach and citrus leaf surfaces by applying ten individual drops (10 µL each) per leaf. A minimum of four replicates were utilized for each *E. coli* cell concentration level and carrier material (leaf surfaces). The *E. coli* suspensions deposited onto the leaf surfaces were air-dried with mild heat application for a minimum duration of 2 h prior to imaging. The steps from sample preparation to imaging are illustrated in Figure 10. The sequence of steps used in the *E. coli* cell preparation in this study was similar to that employed in previous studies [11,40].

### 3.3. Image Denoising

The wavelet transform (WT) is a commonly used method for mitigating noise in both continuous and discrete signals, finding widespread utility across diverse applications [41,42,43,44,45]. WT achieves this by decomposing a signal into localized wave-like oscillations known as wavelets, thus offering a distinct advantage over the Fourier Transform, which often lacks the capability to capture localized frequency information. In contrast to its continuous counterpart, the Discrete Wavelet Transform (DWT) is characterized by a successive decomposition of wavelet coefficients by a factor of 2 at each hierarchical level [46]. In this study, two-dimensional DWT (2D DWT) was employed for denoising fluorescence images captured with the CSI-D+ system using the PyWavelets package (version 1.5.0) [46] in Python (version 3.10.8). Four distinct families of wavelets, namely Biorthogonal (bior), Reverse biorthogonal (rbio), Daubechies (db), and Symlets (sym), were investigated at a decomposition level of 1. Each wavelet transformation yielded two distinct sets of coefficients: “approximate” and “detail”. The Detail coefficients encompassed three categories: horizontal, vertical, and diagonal. The “approximate” coefficients were derived by filtering the original image signal through low pass-low pass (LL) filters, while the detail coefficients were obtained through high pass-low pass (HL), low pass-high pass (LH), and high pass-high pass (HH) filter combinations. Subsequently, these coefficients were utilized for image reconstruction and the extraction of denoised images post 2D inverse WT (IDWT) operations. The quality of denoised images were assessed by comparing them with the original noisy ones using a metric called peak signal-to-noise ratio (PSNR) [45,47,48].

### 3.4. Dataset Preparation

The CSI-D+ system initially captured fluorescence images measuring 256 × 256 pixels in grayscale format with 8-bit depth. Each image contained four leaf plugs for citrus leaves and two for spinach leaves, with each plug containing ten droplets representing the *E. coli* concentration levels. The concentrations of these droplets were 0 CFU/mL (control), 10^5^ CFU/mL, 10^6^ CFU/mL, 10^6.7^ CFU/mL, 10^7^ CFU/mL, 10^7.4^ CFU/mL, 10^7.7^ CFU/mL and 10^8^ CFU/mL (maximum). To facilitate a more detailed examination, each composite image was manually segmented into individual leaf plug images. For each concentration level, eight leaf plugs were prepared for citrus and twelve for spinach (eleven for 10^6^ CFU/mL). This resulted in a total of 64 leaf plugs for citrus and 95 for spinach, each representing the same group of ten droplets but with different *E. coli* concentration levels.

To isolate fluorescent regions, each image underwent background removal using an OpenCV-based preprocessing workflow. The process included grayscale conversion, morphological closing with a 3 × 3 elliptical kernel, and Otsu’s thresholding [49] to segment the foreground leaf plug region. The largest contour in each image was fitted with an ellipse to generate a binary mask, which was slightly dilated to ensure full leaf coverage. Multiplying this mask with the original image produced a clean leaf-on-black background, enhancing contrast for downstream analysis. A wavelet-filtered (IDWT-processed) dataset was then generated from these processed images, in which they were denoised and reconstructed through inverse discrete wavelet transformation for enhanced feature visualization.

Each leaf plug was subsequently divided into four quadrants to ensure detailed capture of distinct features. These quadrants were then subjected to rotational augmentation at 0°, 15°, 30°, and 45°, generating four unique combinations of droplets. Each quadrant, containing approximately two to three droplets, served as the experimental unit in this study. This process expanded each original image into 16 variants (4 quadrants × 4 rotations), resulting in 1024 original images of *E. coli* droplets with citrus and 1584 for spinach leaf background. The resulting images were finally resized to a uniform resolution of 640 × 640 pixels for model training and analysis.

To improve dataset diversity, maintain class balance, and increase dataset size, geometric augmentations (vertical flip, horizontal flip, and random rotation) were applied using Roboflow (Roboflow Inc., Des Moines, USA). Duplicates and noisy samples were then filtered out to ensure dataset quality and uniqueness. After augmentation and data curation, the citrus and spinach leaf datasets each contained a total of 12,000 images, with 1500 images per class. This dataset was referred to as 8classes. Two additional datasets were derived from this dataset. In the second dataset (8classes_4bins), images with similar concentrations were grouped into broader categories: no_*E.coli* (0), low (10^5^), medium (10^6^, 10^6.7^, 10^7^), and hot (10^7.4^, 10^7.7^, 10^8^), resulting in four classes in total. In the third dataset (4classes), images from four specific concentrations were selected for each class: no_*E. coli* (0), low (10^5^), medium (10^7^), and hot (10^8^). The structure of all datasets for spinach and citrus leaves is presented in Table 3.

### 3.5. Training Deep Learning Models

To classify the *E. coli* concentration levels from fluorescence images of leaves, various deep learning models were trained using the supercomputer (HiPerGator) of University of Florida. This system was equipped with an NVIDIA B200 GPU (178.36 GB GPU memory, CUDA capability 10, and 148 multiprocessors). Initially, five different versions (n, s, m, l, x) of the YOLO11 [50] classification model were evaluated. These configurations ranged from lightweight models such as YOLO11n (Nano), optimized for high-speed inference, to more robust and complex versions like YOLO11x (Extra-large). The YOLO11 architecture incorporated several advanced components that enhanced performance. One such component was the C3k2 block (Cross Stage Partial with kernel size 2), which improved computational efficiency without compromising feature extraction capabilities. Another was the SPPF module (Spatial Pyramid Pooling—Fast), which enabled multi-scale feature extraction through pooling at multiple scales. Additionally, the C2PSA block (Convolutional block with Parallel Spatial Attention) improved the model’s ability to focus on salient regions of the image, thereby boosting classification accuracy [51]. Performance evaluations of YOLO11 on the Common Objects in Context (COCO) dataset demonstrated its superiority over previous YOLO versions. For instance, YOLO11n achieved an inference speed of 0.0039 s per image and 256 FPS, outperforming YOLOv8n and YOLOv10n in both speed and computational efficiency [52]. Because of the superior performance over other versions, the YOLO11 variants were trained to classify an image dataset containing leaf surfaces with varying *E. coli* concentrations. Each dataset was split into training (80%), validation (10%), and testing (10%) sets. All variants were trained for 70 epochs with a batch size of 16. The models were optimized using the AdamW optimizer with a learning rate of 0.001 and a momentum of 0.9.

In addition to YOLO11, ConvNeXtBase [53] and EfficientNetB7 [54] models from TensorFlow Keras were employed using transfer learning due to their high accuracy and advanced architectural features with the same datasets. ConvNeXtBase is a convolutional neural network inspired by the design principles of vision transformers, incorporating large kernel sizes and layer scaling while retaining the efficiency of CNNs. This hybrid design enables it to capture long-range dependencies more effectively than traditional CNNs. EfficientNetB7, on the other hand, is based on a compound scaling method that uniformly scales depth, width, and resolution using a neural architecture search approach. It employs fused MBConv blocks to enhance training speed and model generalization. These models served as benchmarks for performance comparison, facilitating an evaluation of trade-offs among computational efficiency, architectural complexity, and classification accuracy. ConvNeXtBase showed strong performance on datasets with complex patterns, while EfficientNetB7 offered a balanced solution for scenarios requiring both speed and precision. For model evaluation, *Precision*, *Recall*, *F1 score*, and validation and testing *Accuracy* were used as performance metrics, as defined in Equations (1)–(4).

(1) $$Precision=\frac{TP}{TP+FP}$$

(2) $$Recall=\frac{TP}{TP+FN}$$

(3) $$F1\;score=\frac{2\times P\times R}{P+R}\;$$

(4) $$\mathrm{Accuracy}=\frac{\mathrm{TP}+\mathrm{TN}}{\mathrm{TP}+\mathrm{TN}+\mathrm{FP}+\mathrm{FN}}$$

The variables *TP*, *TN*, *FP*, and *FN* stand for the number of true positives, true negatives, false positives, and false negatives, respectively.

To highlight the most influential regions, based on how the model classified the *E. coli* concentration levels, Eigen-CAM [55] was applied to the models. Unlike gradient-based methods, Eigen-CAM computed the principal components of the feature maps, providing class-agnostic heatmaps that revealed the most salient regions contributing to the model’s predictions. This approach helped ensure that the models focused on biologically relevant fluorescence patterns rather than background noise. By projecting feature responses onto leading eigenvectors, Eigen-CAM offered a transparent view of the model’s decision-making process. The YOLO-V11-CAM toolkit was used to integrate Eigen-CAM with YOLO11, enabling efficient extraction of feature maps and visualization of activation regions. This interpretability analysis was essential for validating the model’s focus and enhancing trust in its predictions [56,57].

## 4. Conclusions

This study demonstrates the effectiveness of the CSI-D+ system for *E. coli* detection on organic plant surfaces such as citrus and spinach leaves, leveraging deep learning models alongside wavelet-based denoising and data augmentation techniques. The methodology involved preprocessing fluorescence images to enhance feature extraction, followed by extensive experimentation with YOLO11-cls variants (n, s, m, l, x), ConvNeXtBase, and EfficientNetB7 across three datasets (8classes, 8classes_4bins, and 4classes). The results confirmed that YOLO11 outperforms classic deep learning architectures, achieving higher accuracy while maintaining comparable inference times. Particularly, YOLO11s-cls was the best model in terms of accuracy, detection speed, and size, which are crucial for implementation on edge devices.

The YOLO11s-cls model achieved average validation accuracies of 88.40% for citrus and 92.00% for spinach leaves. The average test accuracies were 85.90% for citrus and 92.00% for spinach, indicating consistently strong performance across both leaf types. This model recorded the highest validation accuracy of 95.70% for the 4classes dataset in spinach and the lowest validation accuracy of 83.40% for the 8classes_4bins dataset in citrus. Similarly, the highest test accuracy of 95.90% was observed in spinach for the 4classes dataset, while the lowest test accuracy of 80.60% occurred in citrus for the 8classes_4bins dataset. In comparison, EfficientNetB7 achieved average validation accuracies of 62.78% (citrus) and 66.47% (spinach), and test accuracies of 65.96% (citrus) and 65.93% (spinach). ConvNeXtBase showed average validation accuracies of 57.88% (citrus) and 73.94% (spinach), and test accuracies of 58.55% (citrus) and 74.74% (spinach). These findings highlight the superior and more consistent classification performance of YOLO11s-cls across both leaf types, while EfficientNetB7 and ConvNeXtBase revealed limitations in handling the complexity of fluorescence images. Additionally, the selected YOLO11s-cls model offered an efficient detection speed of 0.011 s per image with a compact size of 11 MB, making it highly suitable for real-time applications.

While the YOLO11s-cls model performed well overall, occasional misclassification occurred between adjacent concentration classes, which is expected due to the logarithmic scaling of CFU counts. Logarithmic increments were used to span the full range from zero *E. coli* presence to the highest concentrations (∼10^8^ CFU/mL), with intermediate levels included to better identify points where fluorescence signal was lost. These adjacent-class misclassifications do not affect the practical outcome, as the primary goal is detecting *E. coli* presence. The differences in classification accuracy between citrus and spinach leaves can be attributed to variations in surface properties and autofluorescence characteristics [58]. During pretest trials, camera gains and exposure times were calibrated for each surface, and leaves with higher moisture content, such as spinach, exhibited stronger autofluorescence under lower gains and shorter exposure times. These variations likely arise from differences in cuticle thickness, wax composition, and reflectance, which influence UV-induced fluorescence. Consequently, camera settings needed to be customized for each produce type, suggesting that real-world applications would require tuning based on the specific plant surface to optimize *E. coli* detection. Future work could explore ordinal classification or regression approaches to better capture the continuous nature of bacterial concentration and reduce adjacent-class confusion

Future work will also focus on enhancing model generalizability by incorporating images captured under varying conditions (e.g., different surfaces, heights, and lighting). Additionally, exploring the deployment feasibility of YOLO11 on edge devices and evaluating transformer-based models like NextViT for improved feature extraction will provide valuable insights for practical applications. In terms of hardware, future studies could include optimizing LED power settings for UV-C exposure to mitigate the effects of excessive fluorescence. This issue was observed during data collection and is particularly important because high surface moisture on fruits and vegetables causes intense fluorescence and signal interference, which adversely affects the device’s ability to accurately detect *E. coli*, ultimately reducing classification accuracy under certain conditions.

The early efforts in this study using handheld sensing devices aim to develop a robust, scalable, and accessible solution for detecting *E. coli* on citrus and spinach leaves. In the future, this approach will be extended to other leafy produce, including lettuce and cabbage. Field tests under laboratory, commercial packing, and farm conditions are also planned to assess the practicality and effectiveness of the method. Applications of these techniques could help reduce incidents of *E. coli* contamination, benefiting farmers and fresh market packers by providing a rapid, accessible alternative to traditional vision-based inspection methods.

## Figures and Tables

**Table 1:** Performance and model size comparison of YOLO11s-cls, EfficientNetB7, and ConvNeXtBase across citrus and spinach leaf datasets with varying *E. coli* concentrations, using independent data partitions and a 0.5 confidence threshold.

Models	Size, mb	Datasets	Validation Accuracy		Test Accuracy	
			Citrus	Spinach	Citrus	Spinach
YOLO11s-cls	11.00	8classes	0.907	0.954	0.910	0.954
		8classes_4bins	0.834	0.850	0.806	0.847
		4classes	0.912	0.957	0.862	0.959
EfficientNetB7	254.70	8classes	0.529	0.588	0.561	0.563
		8classes_4bins	0.629	0.645	0.655	0.662
		4classes	0.726	0.762	0.763	0.753
ConvNeXtBase	338.10	8classes	0.470	0.707	0.460	0.688
		8classes_4bins	0.551	0.698	0.576	0.713
		4classes	0.716	0.813	0.720	0.840

**Table 2:** Precision, Recall, and F1-score of YOLO11s-cls model across datasets containing citrus and spinach leaf samples with varying *E. coli* concentrations.

Datasets	Precision		Recall		F1-Score	
	Citrus	Spinach	Citrus	Spinach	Citrus	Spinach
8classes	0.911	0.955	0.910	0.954	0.910	0.954
8classes_4bins	0.807	0.848	0.806	0.847	0.805	0.847
4classes	0.894	0.961	0.895	0.961	0.894	0.961

**Table 3:** Structure of the datasets used to train, validate, and test the deep learning models for classifying leaf samples with different concentration levels of *E. coli*.

Dataset	Number of Classes	Concentration of the Classes (CFU/mL)	Images/Class	
			Citrus	Spinach
8classes	8	0, 10^5^, 10^6^, 10^6.7^, 10^7^, 10^7.4^, 10^7.7^, and 10^8^	1500	1500
8classes_4bins	4	no_*E. coli* (0), low (10^5^), medium (10^6^, 10^6.7^, 10^7^), and hot (10^7.4^, 10^7.7^, 10^8^)	1500	1500
4classes	4	no_*E. coli* (0), low (10^5^), medium (10^7^), and hot (10^8^)	1500	1500

## Data Availability

Image data is not available for public dissemination due to proprietary restrictions of sponsoring company.
